# Effects of a catechins-enriched diet associated with moderate physical exercise in the prevention of hypertension in spontaneously hypertensive rats

**DOI:** 10.1038/s41598-022-21458-z

**Published:** 2022-10-15

**Authors:** Cristina Del Seppia, Giuseppe Federighi, Dosminga Lapi, Federico Gerosolimo, Rossana Scuri

**Affiliations:** 1 Institute of Clinical Physiology, National Council of Research (CNR), Via Moruzzi, 1, 56124 Pisa, Italy; 2 Department of Translational Research on New Technologies in Medicine and Surgery, University of Pisa, Pisa, Italy; 3 Department of Biology, University of Pisa, Pisa, Italy

**Keywords:** Hypertension, Quality of life

## Abstract

Hypertension represents the main risk factor for the onset of cardiovascular diseases. Pharmacological treatments to control hypertension have been associated with new treatments involving physical activity and/or the intake of natural components (nutraceuticals). We here report the effects produced by a combination of a natural component (catechins) and a moderate exercise program on the development of hypertension in spontaneous hypertensive rats compared with those of each individual treatment. Arterial blood pressure and heart rate were measured with a non-invasive method in 28 rats randomly assigned to four groups: rats subjected to moderate physical exercise; rats with a catechins-enriched diet; rats subjected to moderate physical exercise combined with a catechins-enriched diet; control, untreated-rats left to age. All treatments were applied for 6 weeks. The statistical analysis revealed that the three treatments significantly reduced the weekly increase in arterial blood pressure observed in control rats (SBP, *P* < 0.0001; DBP, *P* = 0.005). However, the reduction of arterial blood pressure induced by combined treatments was not higher than that induced by the single treatment, but more prolonged. All treatments showed strong antioxidative properties. Our data show that physical activity and a diet enriched with catechins individually have an important hypotensive effect, while the association did not produce a higher hypotensive effect than the single treatment, even if it was able to decrease blood pressure for a longer time. These findings have important implications for developing a protocol to apply in novel hypertension prevention procedures.

## Introduction

High blood pressure represents the greatest risk factor for the onset of cardiovascular diseases^1^. According to the World Health Organization, hypertension is responsible for at least 45% of deaths due to heart disease and 51% of deaths due to stroke^2^. Unfortunately, its prevalence and negative impacts on health are growing due to increased life expectancy and other behavioral risk factors, such as poor diet, excessive alcohol consumption, lack of physical activity, excess weight, and exposure to persistent stress^3^. The therapeutic approach is based on pharmacological treatments integrated with lifestyle changes^4^. These approaches are generally effective but have limitations such as their cost^5^ and the fact that a high percentage of the patients does not follow the prescribed therapy or is drug intolerant^6,7^. For this reason, numerous attempts have been made to develop alternative procedures beyond pharmacological treatments and diet, which can stably reduce blood pressure^8^. In particular, it has been observed that the habit of performing a moderate but constant physical activity is associated with a lower incidence of hypertension and is also effective in reducing blood pressure values in hypertensive subjects^9,10^. A regular physical activity is also known to improve not only blood pressure values, but also other cardiovascular functions such as heart rate^11,12^ and peripheral vascular resistance^13,14^. Furthermore, exercise acts on reducing the effects on the sympathetic nervous system^15,16^ and contributes to decreasing the activity of the renin-angiotensin system^17^.

It has also been shown that physical activity improves the vascular endothelial functions^18,19^ whose dysfunction is often associated with an increased blood pressure^20–22^. Several studies have observed that such dysfunctions are due to an oxidative stress deriving from a reduction of nitric oxide production by endothelial cells^23,24^. Furthermore, studies performed in humans and experimental animals have highlighted that oxidative stress is a frequent feature in hypertensive subjects^25–27^.

Consequently, it has been proposed that antioxidant molecules can help in managing blood pressure and in treating hypertension^28^. A number of such antioxidants are found in fruits, vegetables, green tea, coffee, and chocolate and have been observed to have positive effects on the metabolic syndrome, the cardiovascular system, and blood pressure^29–33^. Attention has particularly focused on a special group of antioxidants, the catechins, that are molecules belonging to the category of flavonoids contained especially in teas^34^. Catechins constitute about 80–90% of total flavonoids in green tea, while they represent only 20–30% of total flavonoids in black tea^35^. Catechins administration has been shown to prevent the development of hypertension in the well-known animal model of spontaneously hypertensive rats (SHRs)^36,37^.

The aim of the present work was to evaluate the effects of the combination of a catechins-enriched diet and a moderate exercise program on the development of hypertension in SHRs and to compare them with the action of each individually administered treatment.

## Methods

### Animals and treatments

Male SHRs (Charles River, Calco, Italia) were used. The animals arrived in our lab at the age of 4 weeks, when they were in normotensive conditions. Three days after their arrival in the housing, systolic, diastolic blood pressure (SBP and DBP respectively) and heart rate (HR) were measured (Table 1). In housing, the animals were kept in a controlled environment at a constant temperature (24 ± 1 °C) and humidity (60 ± 5%), subjected to a dark/light cycle of 12:12 h and with food and water ad libitum.

**Table 1:** Parameters measured in the four groups at the arrival in our lab and at the end of the treatments (Means ± S.E.)

	Basal				At the end of the treatment			
	Weight (g)	SBP (mmHg)	DBP (mmHg)	HR (bites/min)	Weight (g)	SBP (mmHg)	DBP (mmHg)	HR (bites/min)
Control	95 ± 1.00	108 ± 1.12	81 ± 3.23	545 ± 4.95	280 ± 8.26	226 ± 1.96	197 ± 4.41	618 ± 29.76
Training	100 ± 1.05	109 ± 0.20	88 ± 2.85	536 ± 7.89	263 ± 7.63	173 ± 1.15	153 ± 4.46	587 ± 14.18
Catechins	93 ± 2.30	110 ± 2.50	82 ± 4.76	546 ± 11.28	275 ± 6.19	168 ± 2.24	150 ± 2.33	514 ± 9.92
Training + catechins	97 ± 1.87	108 ± 1.50	83 ± 1.01	543 ± 2.89	272 ± 3.31	189 ± 2.47	173 ± 4.31	537 ± 28.73

Starting from the 6th week of age, the animals were randomly assigned to four groups: (i) rats subjected to moderate physical exercise for 6 weeks (training, see below, n = 7), (ii) rats with a catechins-enriched diet for 6 weeks (n = 7), (iii) rats subjected to moderate physical exercise associated with a catechins-enriched diet for 6 weeks (training + catechins-enriched diet, n = 7) and (iv) rats left to age without any treatment for 6 weeks (control, n = 7).

The catechins-enriched diet consisted of the administration of an extract of *Malus pumila* Miller cv. Annurca harvested less than 3 months, an apple extremely rich in different phenolic compounds such as catechins (117.6 mg/Kg of fresh weight), epicatechin, a catechin with (2R,3R)-configuration (62.2 mg/Kg of fresh weight), and chlorogenic acid (130.9 mg/Kg of fresh weight)^38^, and with a stronger antioxidant activity than other varieties of apples^38,39^. The extract was produced according to the method of Napolitano et al.^38^ at the University of Naples and kindly provided to us. In our laboratory, the extract was stored at 4 °C, wrapped in aluminum foil to protect it from light and diluted daily in the water drunk by the animals. The extract was administered at the final concentration of 30 mg/kg b.w. and its administration began 2 days earlier than the start of the training protocol and ended on the day of the last training session. This extract concentration was tested through pilot experiments where we tried different dosages 20, 25, 30 and 35 mg/kg b.w. to test the antioxidative effect (see supplementary material) These tests indicated that dosages below 30 mg/kg b.w. were not effective, while higher dosages did not increase the protection obtained with the dose of 30 mg/kg b.w., that was therefore chosen for our experiments.

Throughout the treatment, the amount of water taken in by each rat was evaluated and all animals treated with a catechin-enriched diet ingested a comparable amount of water (about 25 ml/day).

### Ethics declarations

The animals were handled in accordance with the ARRIVE guidelines for the safety and use of laboratory animals (NIH publication no. 68-23 reviewed in 1985).

The experimental protocol was approved by the Local University Ethics Committee and the Italian Ministry of Health (authorization no. 156/2017-PR).

All methods were performed in accordance with the relevant guidelines and regulations.

### Training protocol

Starting from the 6th week of life, SHRs subjected to moderate intensity exercise were trained for 3 sessions a week on alternate days for 6 weeks, for a total of 18 sessions (Table 2). In each training session, the animal was induced to walk following the rotary motion of a motorized wheel. The movement was impressed by a toothed belt connected to an electric stepper motor (US Digital, Washington 98684, USA), driven by a proper computerized system programmed through the LAB VIEW software (National Instruments SRL, Milan, Italy)^40^.

**Table 2:** Training session planning.

Week of training	Monday	Tuesday	Wednesday	Thursday	Friday	Saturday	Sunday
1	5 m/min × 4 min 6 m/min × 6 min 7 m/min × 10 min **20 min**	Rest	5–7 m/min × 6 min 8 m/min × 4 min 9 m/min × 5 min 10 m /min × 5 min 11 m/min × 5 min **25 min**	Rest	5–11 m/min × 5 min 11 m/min × 15 min 9 m/min × 5 11 m/min × 5 min **30 min**	Rest and blood pressure measurement	Rest
2	7–9 m/min × 3 min 10–12 m/min × 2 min 12 m/min × 5 min 13 m/min × 10 min 12 m/min × 10 min **30 min**	Rest	7–9 m/min × 3 min 10–12 m/min × 2 min 12 m/min × 5 min 13 m/min × 10 min 12 m/min × 10 min **30 min**	Rest	7–9 m/min × 3 min 10–12 m/min × 2 min 12 m/min × 5 min 13 m/min × 10 min 12 m/min × 10 min **30 min**	Rest and blood pressure measurement	Rest
3	7–9 m/min × 2 min 10–13 m/min × 3 min 13 m/min × 5 min 14 m/min × 10 min 13 m/min × 10 min **30 min**	Rest	7–9 m/min × 2 min 10–13 m/min × 3 min 13 m/min × 5 min 14 m/min × 10 min 13 m/min × 10 min **30 min**	Rest	7–9 m/min × 2 min 10–13 m/min × 3 min 13 m/min × 5 min 14 m/min × 10 min 13 m/min × 10 min **30 min**	Rest and blood pressure measurement	Rest
4	7–9 m/min × 2 min 10–14 m/min × 3 min 14 m/min × 5 min 15 m/min × 10 min 14 m/min × 10 min **30 min**	Rest	7–9 m/min × 2 min 10–14 m/min × 3 min 14 m/min × 5 min 15 m/min × 10 min 14 m/min × 10 min **30 min**	Rest	7–9 m/min × 2 min 10–14 m/min × 3 min 14 m/min × 5 min 15 m/min × 10 min 14 m/min × 10 min **30 min**	Rest and blood pressure measurement	Rest
5	8–10 m/min × 2 min 11–15 m/min × 3 min 15 m/min × 5 min 16 m/min × 10 min 15 m/min × 10 min **30 min**	Rest	8–10 m/min × 2 min 11–15 m/min × 3 min 15 m/min × 5 min 16 m/min × 10 min 15 m/min × 10 min **30 min**	Rest	8–10 m/min × 2 min 11–15 m/min × 3 min 15 m/min × 5 min 16 m/min × 10 min 15 m/min × 10 min **30 min**	Rest and blood pressure measurement	Rest
6	9–11 m/min × 2 min 12–16 m/min × 3 min 16 m/min × 5 min 17 m/min × 10 min 16 m/min × 10 min **30 min**	Rest	9–11 m/min × 2 min 12–16 m/min × 3 min 16 m/min × 5 min 17 m/min × 10 min 16 m/min × 10 min **30 min**	Rest	9–11 m/min × 2 min 12–16 m/min × 3 min 16 m/min × 5 min 17 m/min × 10 min 16 m/min × 10 min **30 min**	Rest and blood pressure measurement	Rest

Significant values are in bold.

The apparatus consisted of a plastic wheel (diameter 42 cm), closed at the rear and perforated along the entire external surface, so that air and light penetrated inside. At the front, the wheel was closed with a transparent plexiglass panel of the same diameter, which delimited a corridor along which the animal was forced to walk, following the clockwise or anticlockwise rotational movement of the wheel.

**Figure 1:**
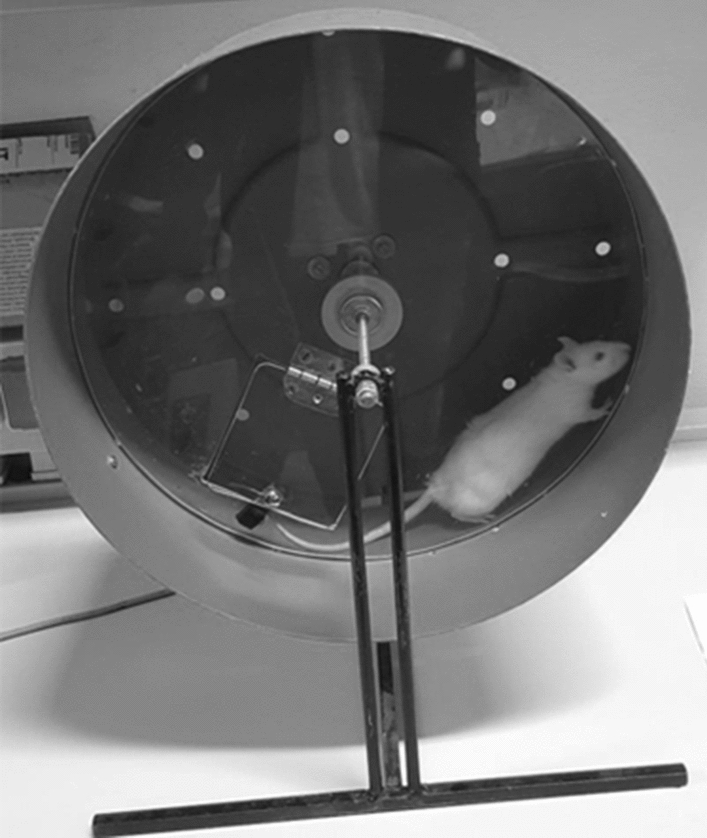
Picture of the training apparatus.

In each session, the animal was taken from the breeding room to the training room, allowed to acclimatize for 10–15 min, and then collected manually from the housing cage and placed inside the training apparatus (Fig. 1).

The operator was able to manage the movement of the wheel, to establish the direction of rotation (clockwise or anticlockwise), the rotation speed (expressed in meters walked/min, m/min), the rotation time (min), and to measure the space travelled (m) in real time.

The acquired data were stored in order to be analyzed off-line.

Before starting the training program, each rat was subjected to a short period of adaptation to the apparatus: it was left free to explore and move inside the wheel, without any connection to the software for about 15 min, then the wheel was moved slowly (4–5 m/min) to allow the rat to coordinate its gait with the rotation speed of the apparatus and establish the direction of rotation of the wheel preferred by the animal. When the rat was able to follow the movement of the wheel, walking continuously for at least 15 min, the training program began.

All training sessions took place between 9:00 and 15:00, to reduce circadian influences and lasted 30 min (except the first two, which lasted 20 and 25 min, respectively). Every week the wheel speed was gradually and steadily increased for all the rats from the initial speed of 5 m/min until they reached 16 m/min at the end of the 18th session.

The training program was structured as reported in Table 2: in the first week, the wheel speed of rotation was progressively increased at each session starting from 5 m/min up to 11 m/min. From the second week onwards, the training protocol was kept constant throughout the week, but the speed was increased every 3 sessions until it reached the value of 16 m/min at the end of the 18th session.

At the end of each training session, the animal was manually placed in its housing cage and brought back to the animal housing until the next session.

**Figure 2:**
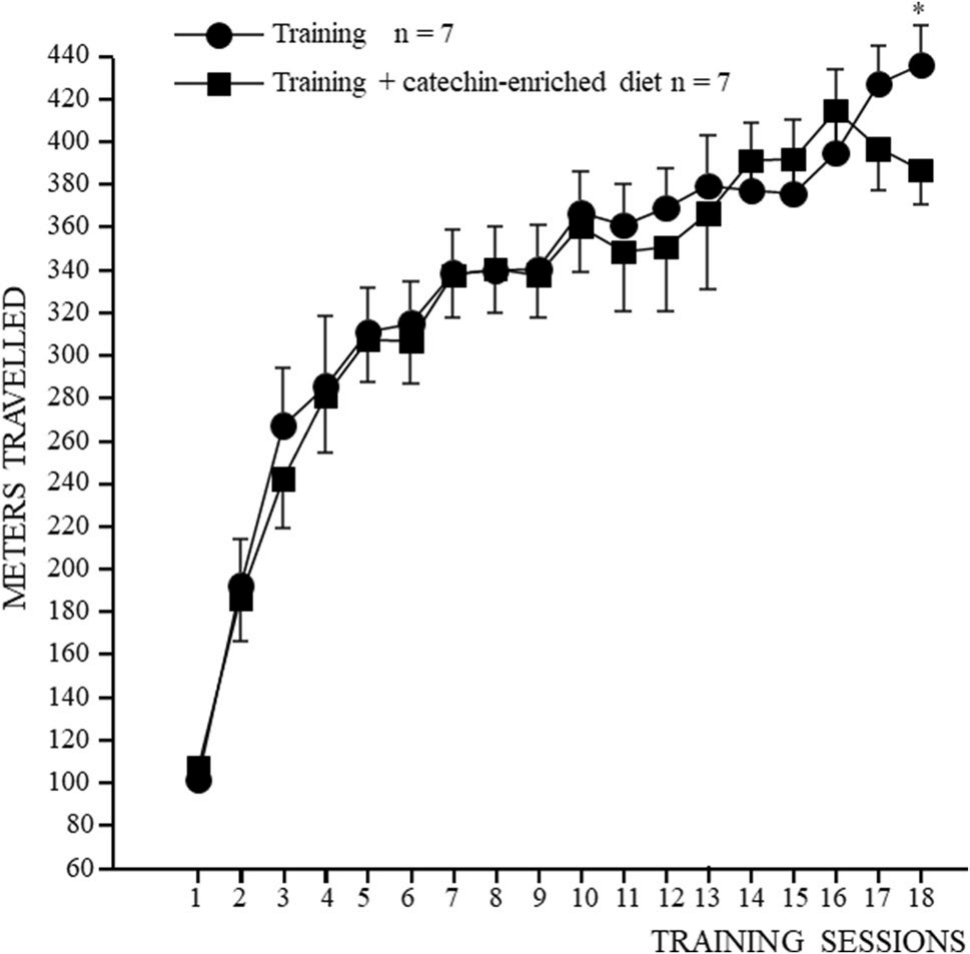
Distance travelled by the trained rats (black circles) and the rats subjected to moderate physical exercise associated with the catechins-enriched diet (black squares) during every test session. *Asterisks* indicate significant differences (*P* < 0.0001) between the two groups in post hoc comparisons after two-way ANOVA for repeated measures (F_17, 72_ = 2.096, *P* = 0.0159).

Figure 2 shows the distance travelled at the end of every training session by trained rats and rats trained and subjected to a catechins-enriched diet. The latter showed signs of fatigue in the last week of training, so we were forced to reduce the increase in wheel speed in order to allow the animals to continue walking rather than stopping for the 30 min required by the training protocol.

### Blood pressure recording

SBP, DBP and heart rate (HR) were measured using a Mouse and Rat Tail Cuff Method Blood Pressure Systems (IITC, Life Science Inc, Los Angeles, CA, USA), which permits the blood pressure to be recorded through a photo-sensor positioned in a cuff that surrounds the animal’s tail. The rats were introduced in a plexiglass tube so that it was maintained in a prone position with the tail inserted in a cuff with the pressure sensor. This was connected to a compressed air cylinder through an arrangement of inlet and outlet valves, so as to allow the cuff to inflate and deflate at a constant speed. The tail blood pressure was continuously recorded by the pressure sensor and frequency and pressure signals were digitized on a desktop computer where they were recorded^41^. Measurements took place once a week (Table 2), and four days after the end of treatment (i.e., in the thirteenth week, when rats were three months old).

In addition, to estimate the myocardial workload we have calculated the rate pressure product (RPP), defined as the product of heart rate (HR) and systolic blood pressure (SBP) and expressed as RPP = SBP × HR/1000^42^

### Evaluation of ROS production

The presence of ROS was detected 4 days after the end of each treatment and in rats just arrived in our housing (young rats) in order to assess the antioxidative properties of each treatment. The content of ROS has been evaluated by using an in vivo fluorometric technique introduced by Watanabe in 1998^43^ utilizing DCFH-DA, a fluorescent hydrogen peroxide sensitive dye, that allows the measurement of intracellular oxidative species such as H_2_O_2_ in the rat cortex neurons. This probe is hydrolyzed inside the cell to DCFH carboxylate that remains trapped in the cell. The oxidation of DCFH leads to the formation of a fluorescent product, 2′-7′-dichlorofluorescein (DCF), that can be monitored with fluorescence microscopy. The fluorescence intensity of DCF is correlated directly to the intracellular reactive ROS level that we evaluated by an appropriate filter (522 nm) and quantified by normalized grey levels) (NGL)^44^. We decided to measure ROS in the brain where we usually evaluate oxidative stress, as hypertension causes serious damages to the brain and the technique we developed is particularly useful in this body district. For experimental convenience, we studied the ROS formation at the level of the pial surface in the parietal area through a closed cranial window whose preparation has been previously described^45^. Local administrations of DCFH-DA was performed mixing the drug with artificial cerebrospinal fluid to obtain a concentration of 250 mM^43^. The solution was superfused over the pial surface for 30 min and 5 and 15 min after we detected ROS formation. The measurements were carried out in anesthetized animals with an intraperitoneal injection of α-chloralose (60 mg/kg b.w.).

### Data analysis

Data were expressed as means ± SEM. Since the data collected were normally distributed according to the Kolmogrov-Smirnov method, the comparisons were performed with parametric tests. One way ANOVA was done to compare ROS formation among the different groups considered.

Two-way ANOVA for repeated measures was used to compare the distances travelled by the trained rats, SBP, DBP, HR and RPP data within each group and among the different groups, considering the time and treatment factors and the interaction "time by treatment". Post hoc tests were performed to analyse the significant differences among the groups at each time considered, using the Tukey’s multiple comparisons test.

These analyses were conducted using GraphPad Prism 7.0 software (Software Inc. San Diego CA).

We also evaluated the rate of variation in blood pressure during the 6 weeks of treatment and 4 days after the end of each treatment by calculating the difference in SBP and DBP with respect to the values measured at the rat’s arrival in our lab (normotensive condition, basal). With SPSS 21.0 package a contrast analysis was done and multiple comparisons were carried out with Bonferroni test.

Statistical significance was set at *P* < 0.05.

## Results

### Antioxidative properties of the different treatments

**Figure 3:**
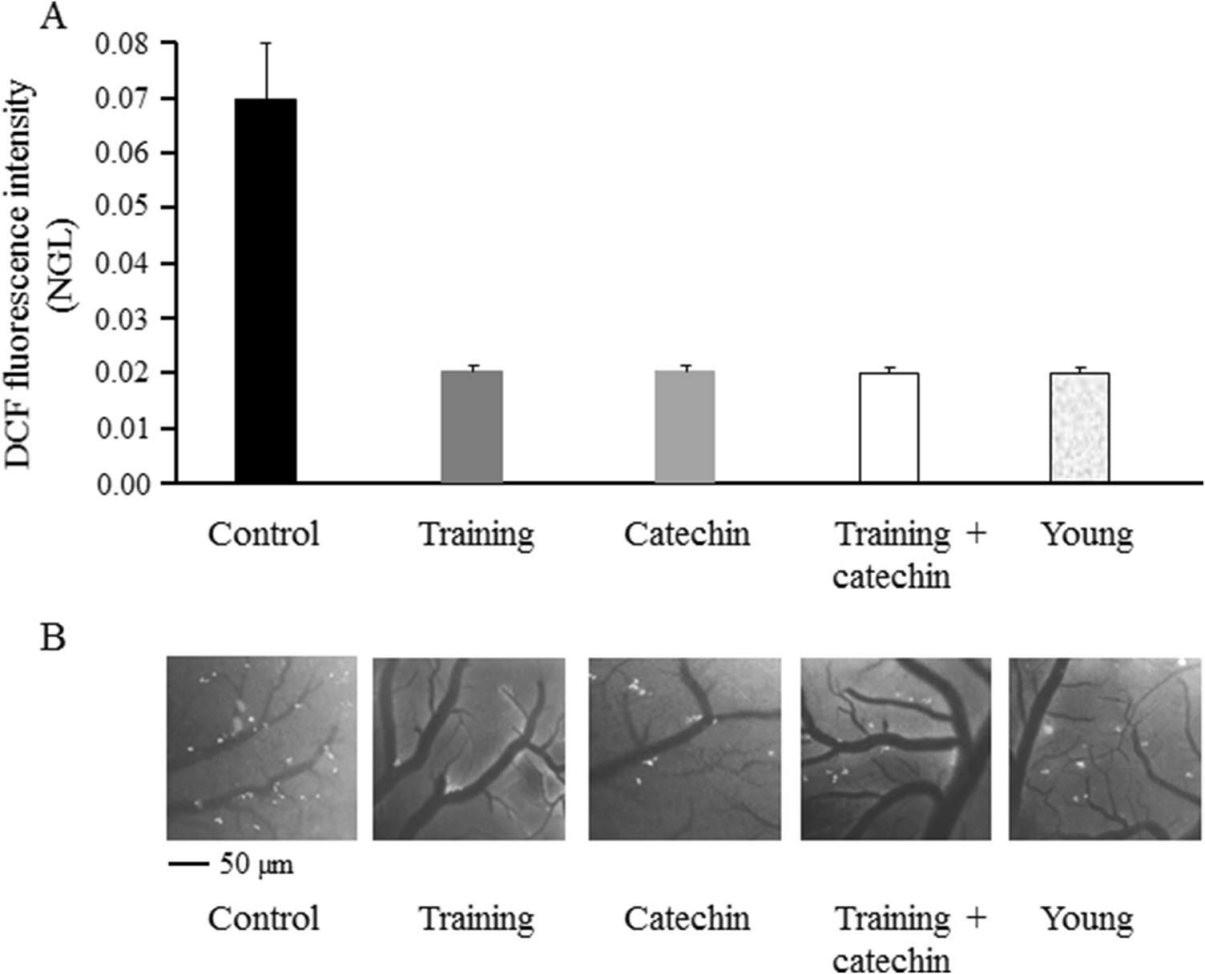
Evaluation of ROS formation in pial microvasculature of the parietal cortex. ROS formation was quantified by NGL (**A**). Computer-assisted images of pial microvascular networks showing fluorescence spots corresponding to the presence of ROS (**B**).

In pial microvasculature of the parietal cortex, control SHRs (i.e. SHRs left to age without undergoing any treatment) showed ROS formation (0.07 ± 0.001 NGL) while in young SHRs (i.e. SHRs just arrived in our lab) ROS formation was significantly much lower (NGL = 0.02 ± 0.001, *P* < 0.001 vs. adult SHRs). Training or catechin-enriched diet or the combined treatment with training and catechin-enriched diet preserved the pial microvasculature by ROS formation. In all three experimental groups subjected to treatment ROS formation was 0.02 ± 0.002 NGL (*P* < 0.001 vs. control, *P* = ns vs. young SHRs) (Fig. 3).

### Effects of the different treatments on the physiological increase of arterial blood pressure and heart rate

A two-way ANOVA for repeated measures was performed by considering the treatment factor (for SBP:F_3,18_ = 692.4, *P* < 0.0001; for DBP:F_3,18_ = 383.7, *P* = 0.005), the time factor (weeks) (for SBP: F_6,36_ = 962.2, *P* < 0.0001; for DBP:F_6,36_ = 767.4, *P* < 0.0001) and the interaction between these two factors (for SBP: F_18,108_ = 100.8, *P* < 0.0001; for DBP: F_18,108_ = 60.35, *P* < 0.0001).

**Figure 4:**
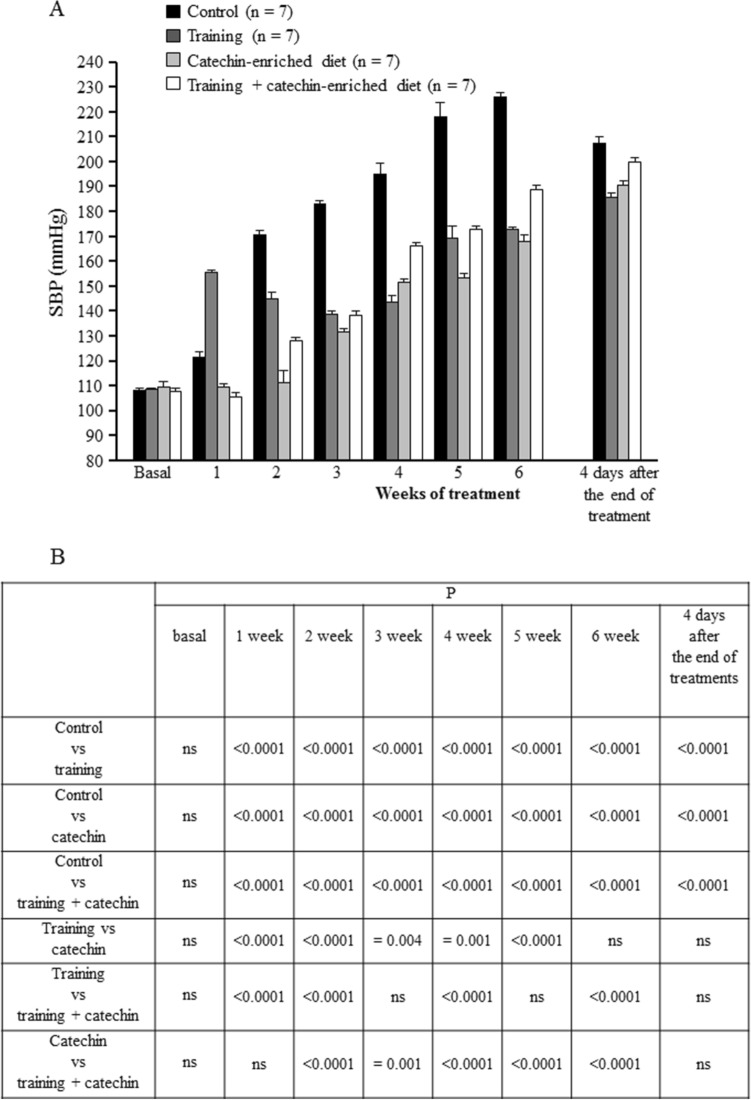
Time-course of systolic blood pressure (SBP, mean ± SEM) in the different groups of rats considered (**A**). Summary of the statistical analysis results (**B**).

**Figure 5:**
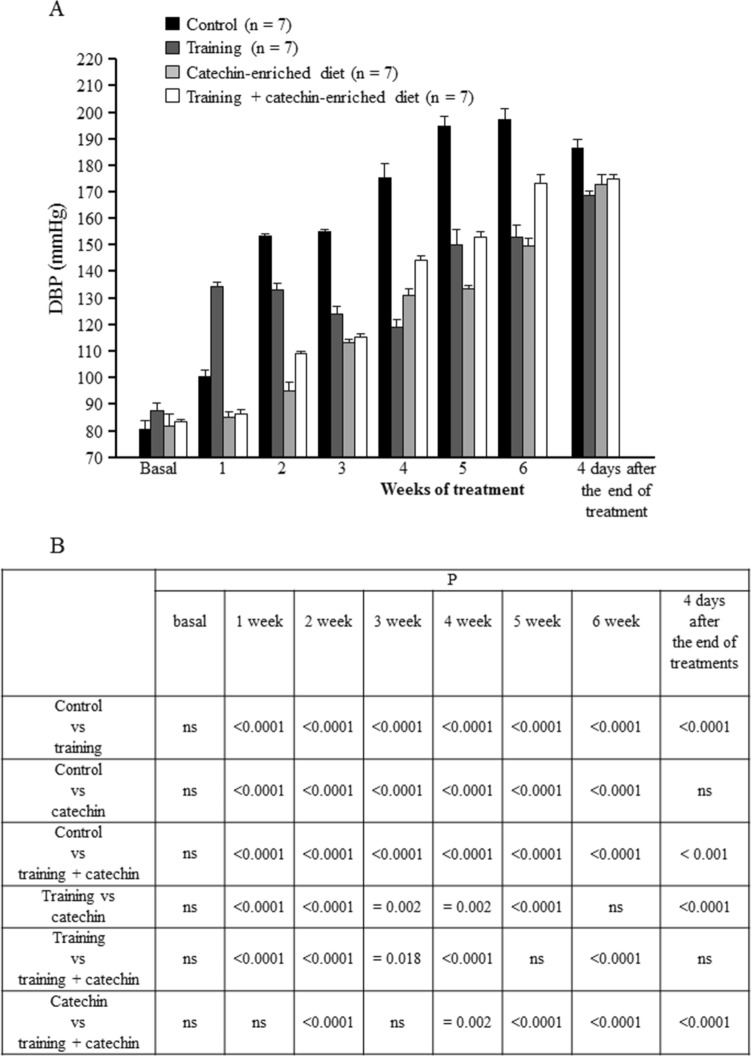
Time-course of diastolic blood pressure (DBP, mean ± SEM) in the different groups of rats considered (**A**). Summary of the statistical analysis results (**B**).

At the arrival in our lab, no significant difference was observed among the animals in SBP, DBP and HR (Figs. 4B,5B and Tables 1,3). As shown in Table 3 any significant effect of treatments on HR was detected.

**Table 3:** Heart rate measured at the arrival in our lab (basal) and at the different times considered.

	HR (bpm)															
	Basal		1 week		2 week		3 week		4 week		5 week		6 week		4 days after the end of treatments	
	Mean	SE	Mean	SE	Mean	SE	Mean	SE	Mean	SE	Mean	SE	Mean	SE	Mean	SE
Control	545	4.95	575	11.33	641	15.88	640	14.23	551	7.12	545	16.31	618	29.76	534	34.92
Training	536	7.89	614	33.48	544	32.53	536	34.56	586	14.81	553	25.46	587	14.18	555	69.33
Catechin	546	11.28	569	31.88	612	45.73	513	58,09	508	27.61	526	21.09	514	29.92	568	34.49
Training + catechin	543	2.89	488	44.30	565	33.28	580	20.46	509	9.45	596	38.4	537	28.73	558	20.66

### Effects of training

Multiple comparisons between the mean values of the control group and trained rats during different weeks, revealed that both SBP and DBP of the training rats were significantly increased in week 1 and reduced in the remaining weeks (*P* < 0.001) with respect to controls, including the measurements taken 4 days after the end of treatment, when a statistically significant difference for SBP and DBP was still observed (*P* < 0.0001). In the control group, blood pressure increased by 118 ± 1.87 mmHg for SBP and 116.25 ± 4.18 mmHg for DBP from the age of 4 weeks (normotensive values) to 11 weeks, while in training rats this difference was reduced to 63.8 ± 1.16 mmHg for SBP and 65 ± 4.78 mmHg for DBP in the same period (Figs. 4,5; Tables 4,5).

**Table 4:** SBP mean changes (mmHg) from basal values at the different times considered.

	1 week		2 week		3 week		4 week		5 week		6 week		4 days after the of treatments	
	Mean	SE	Mean	SE	Mean	SE	Mean	SE	Mean	SE	Mean	SE	Mean	SE
Control	13.75	1.75	62.5	2.04	75.00	0.57	87.13	4.10	110.25	5.29	118.00	1.87	99.38	2.68
Training	46.50	1.40	36.2	2.52	29.89	0.38	34.56	2.94	60.30	5.26	63.80	1.16	76.72	0.85
Catechin	− 0.23	0.10	1.50	3.48	22.00	1.01	41.50	1.16	43.50	1.43	58.00	2.00	80.52	0.54
Training + catechin	− 2.30	2.03	20.02	1.56	30.27	1.08	58.40	0.51	64.80	0.34	80.90	10.86	91.80	0.86

**Table 5:** DBP mean changes (mmHg) from basal values at the different times considered.

	1 week		2 week		3 week		4 week		5 week		6 week		4 days after the of treatments	
	Mean	SE	Mean	SE	Mean	SE	Mean	SE	Mean	SE	Mean	SE	Mean	SE
Control	15.38	2.40	72.25	1.25	74.13	0.99	94.38	5.18	113.88	3.52	116.25	4.18	105.50	3.26
Training	46.40	2.51	45.20	2.30	35.86	3.31	31.12	3.22	62.10	6.88	65.00	4.78	80.82	1.94
Catechin	3.20	1.53	13.13	2.57	31.50	0.93	69.50	1.16	51.63	1.06	67.88	2.08	91.00	2.21
Training + catechin	3.30	2.38	26.02	3.84	32.40	1.61	61.40	1.96	70.10	1.54	90.40	4.05	92.10	1.90

### Effects of catechins-enriched diet

Multiple comparisons between control and experimental rats, highlighted a statistically significant reduction in SBP (*P* < 0.0001) and DBP (*P* < 0.0001) values as early as the first week of catechin administration and throughout the observation period (46 days). A statistically significant reduction in the measurements performed 4 days after the end of treatment was observed for SBP (*P* < 0.0001), but not for DBP (Figs. 4,5).

In rats with a catechins-enriched diet, blood pressure increased from the normotensive values up to 11 weeks by 58 ± 2 mmHg for SBP and 67.88 ± 2.08 mmHg for DBP (Tables 4,5).

### Effects of training + catechins-enriched diet

Multiple comparisons between the mean values of the control group and those of the group of experimental rats, revealed a statistically significant difference between control and training + catechins-enriched diet rats in all weeks (*P* < 0.0001 in all cases). In the measurements recorded 4 days after the end of treatment, the SBP and DBP values of training + catechins-enriched diet rats were significantly lower than in the control group (*P* < 0.001 or lower) (Figs. 4,5).

In training + catechins-enriched diet rats the arterial blood pressure increased from the values recorded in the normotensive condition up to the end of the sixth week of treatment by 80.9 ± 10.86 mmHg for SBP and 90.4 ± 4.05 mmHg for DBP (Tables 4,5).

### After-treatment effects

We performed the Tukey’s multiple comparison test among the different times considered within each experimental group. In particular, we analyzed the blood pressure values between the sixth week of treatment and four days after its end (Figs. 4,5). In the training group both SBP and DBP values showed a significant increase (*P* < 0.001) as was the case for the catechins-enriched diet group (*P* < 0.001). However, SBP and DBP in the training + catechins-enriched diet group did not show any significant difference with respect to the values measured soon after the end of the treatment (SBP, *P* = 0.999; DBP, *P* = 0.998).

In order to compare the increase in SBP and DBP in the SHRs undergoing the different treatments, we performed a contrast analysis of the differences in the values of SBP and DBP recorded at the various times considered with respect to basal values (Tables 4,5 respectively). Two-way ANOVA for repeated measures revealed an interaction time by treatment for both SBP (F_18,144_ = 46.703, Eta-squared = 0.898, α = 1.00, *P* < 0.001) and DBP (F_18,144_ = 29.124, Eta-squared = 0.96, α = 1.00, *P* < 0.001) and the Bonferroni post-hoc test highlighted significant differences as shown in Tables 6 and 7 respectively.

**Table 6:** Results of statistical analysis on SBP increases.

Groups		Mean differences (I–J)	SD	*P*	Confidence interval (95%)	
(I) group	(J) group				Inferior limit	Superior limit
Control vs	Training	31.15	1.44	< 0.001	26.80	35.49
	Catechin	45.60	1.44	< 0.001	41.26	49.94
	Training + catechin	31.73	1.44	< 0.001	27.3°9	36.07
Training vs	Control	31.15	1.44	< 0.001	− 35.49	− 26.80
	Catechin	14.50	1.44	< 0.001	10.11	18.79
	Training + catechin	0.58	1.44	1.000	− 3.76	4.92
Catechin vs	Control	− 45.60	1.44	< 0.001	− 49.94	− 41.25
	Training	− 14.45	1.44	< 0.001	− 18.79	− 10.10
	Training + catechin	− 13.87	1.44	< 0.001	− 18.21	− 9.53
Training + catechin vs	Control	− 31.73	1.44	< 0.001	− 36.07	− 27.39
	Training	− 0.58	1.44	1.000	− 4.92	3.76
	Catechin	13.87	1.44	< 0.001	9.53	18.21

**Table 7:** Results of statistical analysis on DBP increases.

Groups		Mean differences	SD	P	Confidence interval (95%)	
					Inferior limit	Superior limit
Control vs	Training	32.75	1.87	< 0.001	27.13	38.37
	Catechin	38.28	1.87	< 0.001	32.65	43.90
	Training + catechin	31.43	1.87	< 0.001	25.80	37.06
Training vs	Control	− 32.75	1.87	< 0.001	− 358.37	− 27.13
	Catechin	5.52	1.87	= 0.056	− 0.01	11.15
	Training + catechin	− 1.32	1.87	1.000	− 6.94	4.31
Catechin vs	Control	− 38.28	1.87	< 0.001	− 43.90	− 32.65
	Training	− 5.52	1.87	= 0.056	− 11.15	0.01
	Training + catechin	− 6.847	1.87	= 0.013	− 12.47	− 1.22
Training + catechin vs	Control	− 31.43	1.87	< 0.001	− 37.06	− 25.81
	Training	1.32	1.87	1.000	− 4.31	6.94
	Catechin	6.84	1.87	= 0.013	1.22	12.47

**Figure 6:**
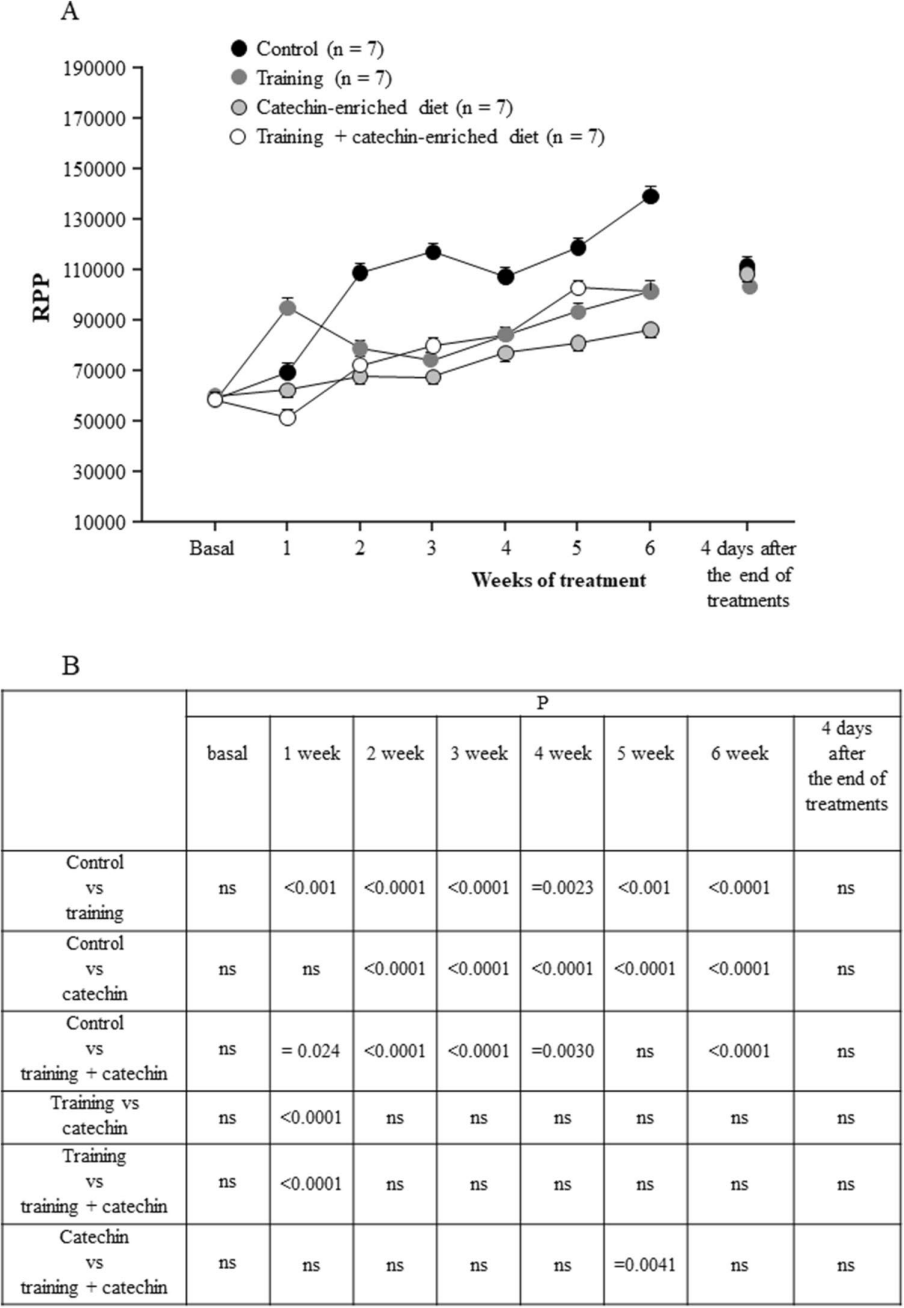
Time-course of rate pressure product (RPP, mean ± SEM) in the different groups of rats considered (**A**). Summary of statistical analysis results (**B**).

Finally, the different treatments were found to have an effect on myocardial workload, estimated through rate pressure product (RPP; Fig. 6). The overall ANOVA revealed a significant main effect of treatments (F_3,12_ = 59.09, *P* < 0.001) and a significant trend for RPP to reduce during the six weeks (F_21,84_ = 5,67, *P* < 0.001). Multiple comparisons are shown in Fig. 6B.

## Discussion

Our data show that physical activity reduces the increase in arterial blood pressure that is normally observed in SHRs starting from the fourth week of life. The treatment modifies the weekly increase in arterial blood pressure values over time, as shown by the differences from baseline values during the six weeks of treatment (Tables 4,5 for SBP and DBP, respectively). From the second week of treatment, the increase in mean pressure in the rats subjected to physical exercise was less than that observed in the control SHR group, which underwent a physiological increase in blood pressure. This effect can be interpreted with the well-known phenomenon called Post Exercise Hypotension (PEH). PEH has been observed in both normotensive and hypertensive humans and spontaneously hypertensive rats but is generally greater in hypertensive subjects^46–50^. The reduction in arterial blood pressure after exercise seems to be due to a decrease in cardiac output and peripheral vascular resistance^50^. The results of our study show that physical exercise reduces the increase of blood pressure that arises with aging in SHRs and that this decrease is related to a reduction in rate pressure product (Fig. 6), which is considered a proxy for the myocardial workload^42^. Various physiological mechanisms appear to be involved in PEH, among which special attention has been paid to the influence of the sympathetic nervous system, which affects various districts including vascular responsiveness (see reviews^10,51^). Vascular resistance can also be modified by locally released molecules from the endothelium, like nitric oxide that induces vasodilation. Several studies have highlighted that in hypertensive subjects the function of the endothelium is altered^22,23,52,53^ and that moderate physical exercise has a beneficial effect by increasing endothelial nitric-oxide synthase, improving the endothelial function and inducing vasodilation^14,18,19,54–56^.

The other two treatments (administration of catechins-enriched diet, and association of catechins-enriched diet and physical activity), had a different effect, causing a reduction in individual arterial blood pressure values shown by treated SHRs over the weeks with respect to controls, without however modifying the rate of increase in blood pressure. These treatments determined, as early as the first week, a hypotensive effect compared to controls (Figs. .4,5), which fall when the treatment was stopped. The effect of catechins-enriched diet is in line with previous studies in which an improvement in blood pressure was observed after administration of antioxidants, both in rats and humans^28,35,36,57–60^.

By comparing the trend of pressure changes over the weeks of treatment it is clear that training slowed down the increase in arterial blood pressure. Treatment with catechins did not modify the rate of the blood pressure increase, but it induced an initial reduction of arterial blood pressure values that was maintained over time, that resulted in lower blood pressure levels over the weeks with respect to controls.

These results suggest that physical exercise constantly modulates the blood pressure regulation centres, not only by preventing the spontaneous tendency of SHRs to increase blood pressure over time, but also by modifying the rate of such an increase. This can be explained by an effect produced by physical activity on the neuronal centres that regulate blood pressure. Differently, the diet enriched with catechins can have an administration-dependent effect, as catechins decrease blood pressure values when are administered, reducing the oxidative stress that is recognized to be involved in many physiological conditions, including physical activity^61^. Our findings additionally show that the association between physical exercise and catechins-enriched diet does not potentiate the hypotensive effect, which turned out to be similar to that induced by the single administration of catechins or by training. As shown in Figs. 4B and 5B, blood pressure values in rats that received combined treatments were significantly higher than those of the other treated groups in 18 out of the 24 comparisons made for SBP and DBP. This shows that the single treatments were somewhat more effective in reducing BP than their combination (which however was still able to reduce it with respect to controls). The positive effect of the combined treatment was observed in the first three weeks of treatment, after which catechin administration becomes unable to prevent the oxidative stress induced by physical activity, with blood pressure consequently increasing during exercise. It is known that, while a moderate physical exercise reduces oxidative stress, acute exercise increases oxidative stress, with negative physiological consequences^62^. Our rats were subjected to increasing levels of physical activity (Table 2), and it is likely that the oxidative stress induced by the intense activity of the last weeks cannot be compensated by the concomitant catechin administration. This is in line with several experimental results showing that the administration of exogenous antioxidants (catechins included) to human subjects performing physical activity does not have positive effects on various physiological parameters^63,64^. It has even been suggested that high doses of antioxidants have adverse effects on the performance of athletes, outweighing their potential beneficial effects^65^. In our case, the combined effect of catechins and an intense physical activity actually worsened the response to the physiological increase of SHR arterial blood pressure and may additionally explain why training alone had in some case a better effect than its combination with catechins.

However, the combination of training + catechins-enriched diet did have a hypotensive effect with respect to controls, that lasted until the end of our observation period. In fact, four days after the end of the administration a significant increase in blood pressure was measured in trained rats and in those with a catechins-enriched diet rats, while the blood pressure of rats subjected to the combined treatments was not significantly different. The reduction in DBP and SBP observed in control rats 4 weeks after the end of the treatment (Fig. 4,5), is puzzling and we have no firm explanation for this effect. We note however that, despite this unexpected decrease, pressure values recorded in treated groups were still significantly lower than those of controls.

In conclusion, our results show that catechins-enriched diet produces hypotensive effects on the arterial blood pressure only during the administration period, in a manner similar to that of moderate physical activity. However, in the group only subjected to training, the beneficial effects on blood pressure tended to decline over time, probably because of the oxidative stress induced by physical exercise, which mitigate the benefits on the cardiovascular system^66^. These side effects of physical activity can be mitigated by the simultaneous administration of antioxidants like catechins, so that the hypotensive effect of physical activity could be prolonged, as is the case of our training + catechins-enriched diet group.

## Supplementary Information

Supplementary Information.

## Data Availability

The datasets used and analysed during the current study are available from the corresponding author on reasonable request.

## Abbreviations

SHRs: Spontaneously hypertensive rats
ANOVA: Analysis of variance
DBP: Diastolic blood pressure
SBP: Systolic blood pressure
SEM: Standard error of the mean
