# Supplementary material for: Effects of a catechins-enriched diet associated with moderate physical exercise in the prevention of hypertension in spontaneously hypertensive rats
Source: Sci Rep. 2022 Oct 15;12:17303. doi: 10.1038/s41598-022-21458-z (PMC9569358; doi:10.1038/s41598-022-21458-z)
Supplement: Supplementary file 1 — Supplementary Information. [file 41598_2022_21458_MOESM1_ESM.docx]

**Table 8 Dose dependent effect of the extract of *Malus pumila* Miller cv. Annurca**

**on ROS content at the level of the pia mater in SHRs**

| **Experimental groups** | **Extract containing catechins dosages**  **(mg/kg b.w.)** | **DCF fluorescence intensity**  **(NGL)** | **Rats**  **N** |
| --- | --- | --- | --- |
| Control | 0 | 0.075 ± 0.010 | 7 |
| Catechin-1 | 20 | 0.084 ± 0.022 | 7 |
| Catechin-2 | 25 | 0.070 ± 0.015 | 7 |
| Catechin-3 | 30 | 0.025 ± 0.012 | 7 |
| Catechin-4 | 35 | 0.020 ± 0.010 | 7 |
